# Emotional response patterns, mental health, and structural vulnerability during the COVID-19 pandemic in Canada: a latent class analysis

**DOI:** 10.1186/s12889-022-14798-y

**Published:** 2022-12-14

**Authors:** Chris Richardson, Trevor Goodyear, Allie Slemon, Anne Gadermann, Kimberly C. Thomson, Zachary Daly, Corey McAuliffe, Javiera Pumarino, Emily K. Jenkins

**Affiliations:** 1 School of Population and Public Health, University of British Columbia, Vancouver, Canada; 2 Centre for Health Evaluation and Outcome Sciences, St. Paul’s Hospital, Vancouver, Canada; 3 School of Nursing, University of British Columbia, T201-2211 Wesbrook Mall, Vancouver, BC V6T 2B5 Canada; 4 Human Early Learning Partnership, School of Population and Public Health, University of British Columbia, Vancouver, Canada

**Keywords:** Mental health, Emotions, Covid-19, Public health, Social determinants of health, Structural vulnerability, Social inequities, Cross-sectional survey, Latent class analysis

## Abstract

**Background:**

The COVID-19 pandemic has contributed to increases in negative emotions such as fear, worry, and loneliness, as well as changes in positive emotions, including calmness and hopefulness. Alongside these complex emotional changes has been an inequitable worsening of population mental health, with many people experiencing suicidal ideation and using substances to cope. This study examines how patterns of co-occurring positive and negative emotions relate to structural vulnerability and mental health amid the pandemic.

**Methods:**

Data are drawn from a cross-sectional monitoring survey (January 22–28, 2021) on the mental health of adults in Canada during the pandemic. Latent class analysis was used to group participants (*N* = 3009) by emotional response pattern types. Descriptive statistics, bivariate cross-tabulations, and multivariable logistic regression were used to characterize each class while quantifying associations with suicidal ideation and increased use of substances to cope.

**Results:**

A four-class model was identified as the best fit in this latent class analysis. This included the most at-risk Class 1 (15.6%; high negative emotions, low positive emotions), the mixed-risk Class 2 (7.1%; high negative emotions, high positive emotions), the norm/reference Class 3 (50.5%; moderate negative emotions, low positive emotions), and the most protected Class 4 (26.8% low negative emotions, high positive emotions). The most at-risk class disproportionately included people who were younger, with lower incomes, and with pre-existing mental health conditions. They were most likely to report not coping well (48.5%), deteriorated mental health (84.2%), suicidal ideation (21.5%), and increased use of substances to cope (27.2%). Compared to the norm/reference class, being in the most at-risk class was associated with suicidal ideation (OR = 2.84; 95% CI = 2.12, 3.80) and increased use of substances to cope (OR = 4.64; 95% CI = 3.19, 6.75).

**Conclusions:**

This study identified that adults experiencing structural vulnerabilities were disproportionately represented in a latent class characterized by high negative emotions and low positive emotions amid the COVID-19 pandemic in Canada. Membership in this class was associated with higher risk for adverse mental health outcomes, including suicidal ideation and increased use of substances to cope. Tailored population-level responses are needed to promote positive coping and redress mental health inequities throughout the pandemic and beyond.

## Introduction

Emerging research illustrates that the COVID-19 pandemic has contributed to increases in negative emotions such as fear, worry, loneliness, and irritability [1–4]. Emotions are reflections of inner mental states and can be understood as patterned responses to events or stimuli that can initiate physiological and behavioural changes [5, 6]. There is ongoing debate as to which particular states constitute an emotion and about whether emotions can be dichotomously classified, for example as “positive” and “negative” [7]. Indeed, all emotions contain some degree of both negativity and positivity and can more aptly be characterized as manifesting along a spectrum [7]. Still, there is general agreement that some emotions are experienced as more positive or resourceful and others as more negative or challenging [5, 8]. Negative emotions in particular are highly interconnected with mental health challenges, with negative emotional experiences and individual reactions to one’s own emotions associated with mental health conditions such as depression and anxiety [9, 10]. In the COVID-19 pandemic context, research has underscored that increases in negative emotions (e.g., fear, worry) are contributing to adverse mental health impacts including stress, anxiety, and depression [1, 3]. Alongside these far-reaching emotional and mental health impacts is a concerning escalation in population-level experiences of suicidal ideation [11] and using substances as a way to cope [12, 13].

While negative emotions are associated with poor mental health and mental health challenges, research into positive emotional experiences suggests that positive emotions can support mental health and well-being, even in the context of challenging events or circumstances [14–16]. For example, the broaden-and-build theory holds that experiences of positive emotions enhance capacity for engaging in a wide range of coping strategies in response to stress, and as such, promote well-being, build resiliencies, and protect against mental health challenges [17–19]. This theory has been used to examine associations between positive emotions and coping and resilience in disaster and crisis situations, such as the 9/11 attacks in the United States [20] and the 2009 H1N1 pandemic [21]. Experiencing positive emotions is also integral to overall positive mental health, an affective state that includes qualities such as good self-esteem, the ability to effectively manage stress, and an overall sense of wellbeing [22, 23]. As with negative emotions, positive emotions are felt by – and can also benefit – people across the continua of mental health and illness.

Since the emergence of COVID-19, research on the psychosocial impacts of the pandemic has predominantly focused on the increasing prevalence of negative emotions and decreasing positive emotions among the general population, with limited exploration of the more complex interrelationships between positive and negative emotions [1, 4]. This is concerning given that heightened levels of negative emotions and diminished levels of positive emotions are known to contribute to potentially harmful coping responses and other adverse psychosocial consequences [4], including increased substance use [24–26] and suicidal ideation [27–29], each of which can pose immediate and long-term risks with respect to an individual’s quality of life, safety, and overall health and wellbeing. Although some studies have demonstrated the presence of positive emotions (e.g., optimism, thankfulness) amid the pandemic [30] and examined resilience and coping with pandemic-related stress [31, 32], further inquiry into patterns of co-occurring positive and negative emotions is needed to inform opportunities for prevention and early intervention from a population mental health standpoint. This is particularly so given that many studies examining emotional responses to the COVID-19 pandemic are limited in the sense that they draw from online data sources such as Google searches and Twitter posts, which lack contextual socio-demographic information, precluding analyses of how emotional response patterns may differ within and between socio-demographic groups [30, 33, 34]. With this literature gap, there is demand for targeted studies investigating how pre-existing health and social inequities may lend some populations to be more at risk for precarious emotional response patterns and associated social and mental health sequelae in the context of COVID-19, including to inform responsive policy and practice action [35, 36].

The pandemic has contributed to emotional challenges and adverse mental health impacts among the population at large, though these consequences are not being distributed equally [37]. For example, in Canada, where the current study is set, prior research in the context of the COVID-19 pandemic has identified significant and widening mental health inequities among populations experiencing structural vulnerabilities [38]. Here, the concept of “structural vulnerability” denotes an elevated and unjust risk for adverse health outcomes resulting from an individual’s or group’s interface with overlapping socio-economic, political, and normative power hierarchies, which constrain access to determinants of good health [39]. Available research indicates that people experiencing structural vulnerabilities are being especially hard hit by the psychosocial impacts of COVID-19 and associated public health protections, such as school/workplace closures and social distancing and isolation measures. This includes, for example, people who are younger, have lower household incomes, have pre-existing mental health conditions, and/or experience forms of historical and structural oppression along axes of race/ethnicity, class, gender, sexuality, and ability [37, 38, 40–42]. The extant literature offers broad, high-level understandings of the distribution of emotional responses and related mental health impacts of the pandemic across population subgroups [2, 3], though further evidence is needed about how these emotional responses and related mental health outcomes may be variously associated with structural vulnerability. Such understandings can support targeted public health responses to mitigate the mental health risks associated with negative emotions, while also bolstering positive emotions and coping amid the pandemic, including among inequitably impacted groups.

The aim of the current investigation is to examine how patterns of co-occurring positive and negative emotions relate to mental health outcomes and structural vulnerability in the context of the COVID-19 pandemic. The interconnected research objectives are to: (1) identify patterns of positive and negative emotional responses to the COVID-19 pandemic; (2) characterize each of the emotional response pattern groups according to their socio-demographic profiles and mental health-related coping; and (3) examine the extent to which emotional response patterns to the pandemic are associated with the specific self-reported mental health impacts of suicidal ideation and increased use of substances to cope.

## Methods

### Survey development and approach

The development of this cross-sectional monitoring survey, *“Assessing the Impacts of COVID-19 on Mental Health*,*”* represents a collaboration between academic researchers working in partnership with national mental health advocacy organizations, the Canadian Mental Health Association and the United Kingdom’s Mental Health Foundation. These partnerships provide direct linkages to policy decision makers and have been used to advocate rapid, data-driven policy and programming responses to population mental health during the COVID-19 pandemic.

Survey items were initially developed in March 2020 by the Mental Health Foundation. Original item development was guided by research evidence on mental health impacts of past epidemics and pandemics as well as input from people with lived experience of mental health conditions, gathered via a participatory citizens’ jury and targeted literature reviews [43]. This collaborative, evidence-informed process sought to develop and refine measures of mental health, emotions, and coping strategies that would hold relevance for informing public health policy and practice in the pandemic context. The overarching aim here was to be comprehensive and pragmatic, not exhaustive. The survey was made available in English and French to supports its use in Canada. Further, survey items were modified or added to support identification of disproportionate impacts of the pandemic on groups experiencing increased risks due to structural vulnerabilities and pre-existing inequities. This was achieved by including items on age, ethnicity, race, gender, LGBTQ2 + (lesbian, gay, bisexual, transgender, queer, Two-Spirit, and other sexual and gender minority) identity, socioeconomic status, and mental health and disability status.

### Data collection

Cross-sectional online surveys were distributed by a national polling vendor, Maru/Matchbox, which manages an online “restricted access” panel of approximately 125,000 members (Maru Voice Canada panel). This panel is available to trusted research partners seeking to ensure sample integrity and data quality. Panel participants were recruited using mechanisms to promote inclusion of traditionally under-represented populations (e.g., older adults, racialized persons) and to yield a nationally representative sample according to certain socio-demographics. This involved random sampling of panel participants 18 years or older living in Canada (all provinces/territories) while stratifying selection based on Canadian Census-informed socio-demographics characteristics (age, gender, household income, region) and adjusting for response propensity. The response rate for panel members invited to participate in the survey was 36%.

The current investigation focuses on online survey data collected between January 22–28, 2021, representing the third round of a repeated cross-sectional monitoring survey study [38]. Focusing on this survey round allowed us to undertake a detailed assessment of population-level emotional response patterns and mental health at a particular moment in the pandemic. This was a time of rising COVID-19 case numbers following the winter holidays, associated with which were increasingly restrictive public health protections in provinces and territories across Canada, including advisements against travel, the continued roll-out of vaccine passports (required for many social activities), and school closures and restrictions on social gatherings [44, 45].

### Measures

#### Socio-demographic factors

The measures used in this survey are described in detail elsewhere [46, 47]. Key socio-demographic characteristics assessed included age, gender identity, LGBTQ2 + identity, highest level of education completed, household income, employment status, race/ethnicity, urban/rural place of residence, and being a parent/guardian of a child under 18 years of age. Age was assessed in years and later stratified into three categories: 18–34 years, 35–54 years, and 55 + years. Gender was assessed by asking, “Which gender do you most identify with?” with response options: “Female”; “Male”; “Non-binary”; “Two-Spirit”; “Not listed”; and “Prefer not to answer”. Participants were also asked to indicate their sex assigned at birth, with binary response options “Female” and “Male”. As with our previous work [46], we used comparisons between measures of current gender and sex assigned at birth to identify transgender participants (i.e., if a participant identified their current gender as “Male” and reported being assigned “Female” at birth, we classified them as a trans man). We also identified LGBTQ2 + participants using the question, “Do you identify as being LGBT2Q + (lesbian, gay, bisexual, trans, Two-Spirit, queer, etc.)?”. Race/ethnicity was measured by asking participants to identify their “ethnic origin”. Participants who identified only European origins were classified as non-racialized, those who identified one or more non-European origins were classified as racialized persons, and those who identified Indigenous origins were classified as Indigenous, regardless of other reported origins. Table 1 provides additional detail regarding classification of socio-demographics for this study.

**Table 1:** Socio-demographic description of respondents to a Canadian survey regarding mental health amid COVID-19, 2021

	N	(%)
Age group		
18–34 years	403	13.4
35–54 years	1225	40.7
55 + years	1381	45.9
Gender Identity^1^		
Cisgender man	1464	48.7
Cisgender woman	1505	50.0
Trans man	3	< .1
Trans woman	14	.5
Non-binary	12	.4
Two-Spirit	1	< .1
Not listed	2	< .1
LGBTQ2 + identity (yes or unsure) ^1^	222	7.4
Parent/guardian of a child under 18 years of age	596	19.8
Household income, CAD^1^		
Under $25 k	227	7.5
$25 k- < $50 k	502	16.7
$50 k- < $100 k	1019	33.9
$100 k +	1171	38.9
Education completed		
High school or less	451	15.0
Some college or university	473	15.7
College or university graduate	2085	69.3
Race/ethnicity^1^		
Non-racialized	2113	70.2
Racialized (non-Indigenous)	673	22.4
Indigenous	94	3.1
Urban/rural		
Urban	2314	76.9
Rural	695	23.1
Pre-existing mental health condition^1^		
Yes	503	16.7
No	2481	82.5
Total	3009	100.0

^1^ A small number of respondents chose not to answer some individual sociodemographic questions, which reduced the total counts for these variables

#### Emotions

Participants were asked “Which of the following emotions have you felt as a result of the COVID-19 pandemic in the past 2 weeks? (Please select all that apply)” and were presented with a checkbox list of emotions that included negatively oriented emotions (anxious, stressed, lonely, sad, depressed, hopeless, angry, bored and afraid) and positively oriented emotions (hopeful, secure, comfortable, calm, and empathetic) [47]. These emotions were selected based on the aforementioned citizens’ jury process and original survey upon which the current study extends and are not intended to fully represent all aspects of emotional positivity and negativity.

#### Mental health-related characteristics

Participants were asked if they had a pre-existing mental health condition (“*Yes*,” “*No*,” or “*Prefer not say*”). Sleep quality was assessed by asking, “*During the past 2 weeks, how would you rate your sleep quality overall?*” with response options of “*Very good*,” “*Fairly good*,” “*Fairly bad*” and “*Very bad*.” Overall coping was assessed through the question: *“Overall, how well do you think you are coping with stress related to the COVID-19 pandemic?”* with response options “*Not very well”* and *“Not well at all”* classified as *“Not well,”* and responses of *“Very well”* and *“Fairly well”* classified as *“Well.”* Current mental health was assessed by asking, *“In general, would you say your mental health is:”* with response options for *“Excellent,” “Very good,” “Good,” “Fair”* and *“Poor.”* Self-reported changes in mental health was assessed by asking, *“Compared to before the COVID-19 pandemic and related restrictions in Canada, how would you say your mental health is now?”* with the responses *“Same” or “Better”* combined into “*Not worse*” category, and *“Slightly worse now”* and *“Significantly worse now”* classified as experiencing *“Worse”* mental health*.*

#### Main outcomes

Suicidal ideation was assessed by asking participants, “Have you done or experienced any of the following as a result of the COVID-19 pandemic in the past 2 weeks?” with an option to select “Experienced suicidal thoughts/feelings.” Increased use of substances to cope was assessed by asking participants “Has your use of substances increased as a way to cope at any point during the pandemic?” with the response options “Yes,” “No,” and “Prefer not to say.”

### Statistical analyses

Descriptive statistics were used to describe the socio-demographic characteristics of the sample. Latent class analysis [48] with robust maximum likelihood estimation was then used to identify the optimum number of emotional response patterns (i.e., classes) to the COVID-19 pandemic based on endorsement of negative emotions (anxious, stressed, lonely, sad, depressed, hopeless, angry, bored and afraid) and positive emotions (hopeful, secure, comfortable, calm, and empathetic). This began with a solution containing 2 classes and included evaluating models with an increasing number of classes up to a 6-class solution. The number of classes retained in the final model was based on the overall interpretability of the solution in addition to the Akaike information criterion (AIC), Bayesian information criterion (BIC), sample size-adjusted BIC (SSABIC), entropy, and the Lo-Mendel-Rubin likelihood ratio test (LMR-LRT). Smaller values for BIC, SSABIC, and AIC are indicators of better model fit while higher values are preferred for model entropy [49]. After assigning participants to their most likely class membership, descriptive statistics and Chi-square analyses were used to examine the socio-demographic and mental health related characteristics of each class in the selected latent class model. Separate multivariable logistic regression models were then used to quantify the extent to which class membership was associated with increased use of substances to cope and suicidal ideation after adjusting for key socio-demographic characteristics. Descriptive statistics and logistic regression analyses were conducted with SPSS Version 27 [50] and the latent class analysis was conducted with Mplus Version 7.4 [51].

### Ethics

Ethical approval for this study was provided by the University of British Columbia Behavioural Research Ethics Board (#H20-01,273). All participants provided informed consent online prior to beginning the survey and received a small honorarium through Maru/Matchbox.

## Results

A total of 3034 respondents participated in this January 2021 survey. Of these 3034 participants, 25 were not included in the analyses because they indicated “*Prefer not to answer*” (*n* = 11) or “*Don’t know*” (*n* = 14) to the emotions survey measure. This resulted in a final sample size of 3009 which is characterized in Table 1.

The results of the latent class analysis of the individual emotion responses starting with a solution containing 2 classes and increasing up to a 6-class solution are presented in Table 2. The 4-class solution was selected as the optimal solution because it had the highest entropy and had a solution containing distinct response profiles that could be clearly and pragmatically interpreted. It is important to note that, although the entropy value of 0.748 for the 4-class solution was highest, this level is somewhat lower than the commonly used threshold of 0.80. This indicates that the overall classification accuracy of the solution (i.e., the extent to which the classes are distinct) is on the low side. Additional support for the 4-class solution lies in the shift from a 4- to a 5-class solution being associated with a non-significant improvement in fit according to the Lo-Mendel-Rubin likelihood ratio test (See Table 2 for details). Although there were some relatively small improvements in model fit (i.e., reduced AIC and BIC) associated with going beyond 4 classes, this resulted in a drop in entropy below 0.70.

**Table 2:** Latent class model fit statistics for 2 through 6 class solutions

Number of classes	AIC	BIC	Adjusted BIC	Entropy	Lo-Mendell-Rubin Adjusted LRT Test (*P*-value)
2	40,386.783	40,561.054	40,468.910	0.732	
3	39,444.207	39,708.619	39,568.814	0.717	0.0000
4	39,071.607	39,426.159	39,238.694	0.748	0.0001
5	38,941.799	39,386.492	39,151.365	0.689	0.1111
6	38,815.493	39,350.326	39,067.538	0.677	0.0076

*AIC* Akaike information criterion, *BIC* Bayesian information criterion

**Fig. 1:**
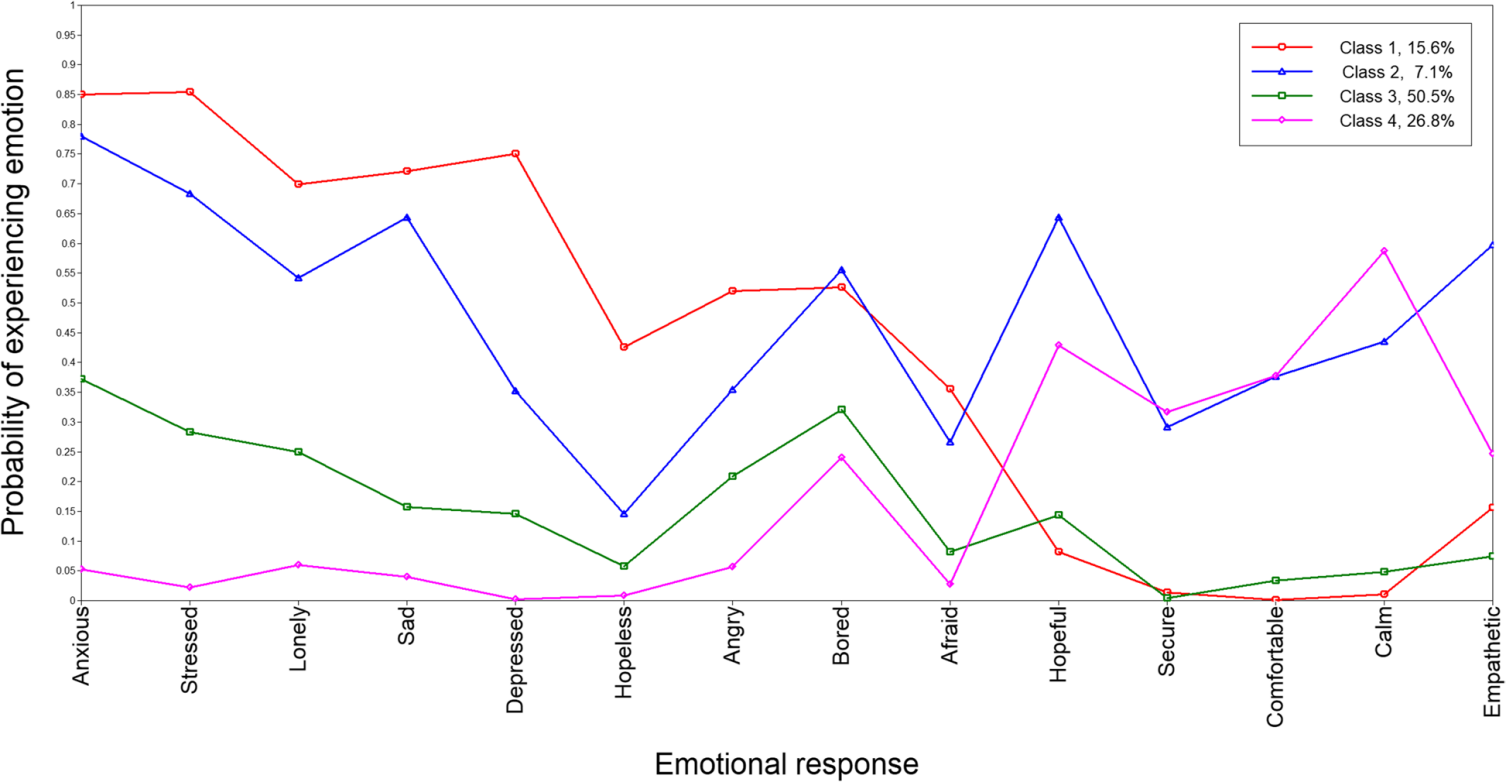
Class specific probabilities of experiencing specific emotions for the 4-class solution

A plot of the class specific probabilities of experiencing each emotion for the 4-class solution is presented in Fig. 1. Class 1 contained 15.6% of the sample and was interpreted as the most at-risk class due the high probability of endorsing negative emotions combined with a low probability of endorsing positive emotions. Class 2 contained 7.1% of the sample and was characterized as mixed risk as it also had high probabilities of endorsing negative emotions (though not quite as high as Class 1) yet had much higher probabilities of endorsing positive emotions. Class 3 contained 50.5% of the sample and had moderate probabilities of endorsing negative emotions and low probabilities of endorsing positive emotions. The large size and response pattern led to the interpretation of this class as the norm or reference class. Class 4 contained 26.8% of the sample and was interpreted as being the most protected class as its members had very low probabilities of reporting negative emotions and high probabilities of reporting positive emotions.

To further characterize the latent classes, the results of separate bivariate (i.e., Chi-square) analyses of socio-demographic features and mental health-related characteristics by class are presented in Table 3. From this table it can be seen that Class 1 (most at risk) tended to have a greater proportion of younger participants (aged 18–34 years: 16.6%) and fewer older participants (aged 55 + years: 38.4%) compared to Class 3 (norm/reference) and Class 4 (most protected). Class 1 also contained a greater proportion of participants in the lower income categories and fewer in the high-income categories compared to the other classes. Class 1 and Class 2 (mixed risk) also had greater proportions of cisgender women and LGBT2Q + members compared to Class 3 and Class 4. With regards to employment, Class 3 had the highest proportion of members who were currently working (57.0%) and Class 4 had the highest proportion of members who were retired (43.1%). Class 1 also had the highest proportion reporting poor sleep (62.0%) and Class 4 had the lowest (12.8%). In addition to socio-demographic characteristics, class membership was also associated with mental-health related characteristics. For example, Class 1 had the highest proportion of members with a pre-existing mental health condition (37.4%) while Class 4 had the lowest (7.6%). Class 1 also had the highest proportion of participants reporting that they were not coping well (48.5%), had fair or poor mental health (54.2%), and had experienced worsening mental health (84.2%). In contrast to Class 1, the proportions for Class 4 were substantially lower than all other classes with respect to the proportion of participants reporting that they were not coping well (1.5%), had fair or poor mental health (1.6%), and had experienced worsening mental health (9.1%).

**Table 3:** Socio-demographic and mental health-related characteristics by latent class

	Class 1 (most at risk) (*n* = 469)	Class 2 (mixed risk) (*n* = 214)	Class 3 (norm/ reference) (*n* = 1519)	Class 4 (most protected) (*n* = 807)	Total (*n* = 3009)
Age (years) **					
18–34 (%)	16.6	17.3	13.4	10.5	13.4
35–54 (%)	45.0	38.3	44.6	31.6	40.7
55 + (%)	38.4	44.4	42.1	57.9	45.9
Income**					
< $25 k (%)	13.4	8.3	6.4	7.0	7.8
$25 k-$50 k (%)	20.2	11.7	17.7	16.1	17.2
$50 k-$100 k (%)	31.1	33.0	34.9	37.5	34.9
$100 k + (%)	35.3	47.1	41.0	39.4	40.1
Gender**					
Cisgender woman (%)	60.6	59.3	48.5	44.4	50.0
Cisgender man (%)	38.0	39.3	50.8	53.4	48.7
Trans identities^1^ (%)	1.5	1.4	.8	2.2	1.3
LGBT2Q + **					
Yes/unsure^2^ (%)	10.4	9.8	7.4	5.1	7.4
No	89.6	90.2	92.6	94.9	92.6
Race/ethnicity**					
Non-racialized (%)	75.7	79.0	71.9	73.2	73.4
Racialized (non-Indigenous) (%)	19.0	17.6	25.0	24.5	23.4
Indigenous (%)	5.3	3.3	3.1	2.4	3.3
Employment^3^					
Working (full or part time) (%)**	49.5	52.8	57.0	46.7	52.8
Student (full or part time) (%)NS	3.0	0.9	1.8	1.4	1.8
Retired (%)**	23.7	26.2	28.7	43.1	31.6
Unemployed (%)**	7.5	13.1	4.9	3.3	5.5
Parent/Guardian (< 18) (%)**	17.9	25.2	22.1	15.2	19.8
Pre-existing mental health condition (%)**	37.4	27.7	13.9	7.6	16.9
Sleep quality**					
Fairly good or very good	38.0	68.1	70.4	87.2	69.7
Fairly bad or very bad	62.0	31.9	29.6	12.8	30.3
Overall coping**					
Well	51.5	88.0	87.8	98.5	84.9
Not well	48.5	12.0	12.2	1.5	15.1
Current mental health**					
Good, very good or excellent	45.8	85.0	85.6	98.4	82.8
Fair or poor	54.2	15.0	14.4	1.6	17.2
Deterioration in mental health**					
Same or better	15.8	42.5	59.4	90.9	59.8
Worse	84.2	57.5	40.6	9.1	40.2

^1^ This includes participants who identified as trans women, trans men, non-binary, and Two-Spirit

^2^ Participants who indicated “unsure” when asked if they identified as LGBTQ2 + were included here to capture individuals who may be questioning their sexual orientation and/or holding fluid sexual identities

^3^ Categories of employment represent separate independent variables and are not mutually exclusive

^**^ *P* < 0.01, NS = not significant

As a first step in examining the relationship between the patterns of emotional responses and main outcomes, we examined the extent to which the latent classes of emotional responses were associated with self-reported mental health impacts of the pandemic in the form of suicidal ideation and increased use of substances to cope. Similar trends to the mental health-related characteristics were observed for suicidal ideation and increased use of substances to cope. More specifically, there was a significant bivariate association between suicide ideation and latent class (Chi-square_(3)_ = 209.88, *p* < 0.01) with the following rates of endorsement: Class 1 (21.5%), Class 2 (6.1%), Class 3 (4.3%), and Class 4 (2.0%). There was also a significant bivariate association between reporting an increased use of substances to cope and class membership (Chi-square_(3)_ = 165.50, *p* < 0.01) with the following rates of endorsement: Class 1 (27.2%), Class 2 (20.4%), Class 3 (9.9%), and Class 4 (4.5%). These associations and the results of separate multivariable logistic regression models quantifying the extent to which class membership is associated with suicidal ideation and increased use of substances to cope – adjusting for key socio-demographic characteristics – are presented in Table 4. From this table, it can be seen that compared to the norm or reference class (Class 3), members of the most at-risk class (Class 1) were significantly more likely to report suicidal ideation (OR = 4.64, 95% CI = 3.19–6.75) and increased use of substances to cope (OR = 2.84, 95% CI = 2.12–3.80) while members of Class 4 were significantly less likely to report both experiencing suicidal ideation (OR = 0.50, 95% CI = 0.28-0.92) and increased use of substances to cope (OR = 0.45, 95% CI = 0.30–0.67).

**Table 4:** Results of separate logistic regression analyses examining the association between class membership and suicidal ideation (Model 1) and reported increases in the use of substances to cope (Model 2) after adjusting for key socio-demographic characteristics

	Model 1: Suicidal ideation			Model 2: Substances to cope		
	Odds Ratio	95% C.I. for Odds Ratio		Odds Ratio	95% C.I. for Odds Ratio	
		Lower	Upper		Lower	Upper
Age: 55 + (Ref)						
Age: 18–34 years	4.32^**^	2.61	7.15	2.95^**^	2.07	4.20
Age: 35–54 years	2.67^**^	1.75	4.07	1.71^**^	1.29	2.27
Household income: $100 k + (ref)						
Household income: < $25 k	1.47	.86	2.51	.65	.41	1.06
Household income: $25 k-$50 k	1.35	.85	2.14	.87	.61	1.25
Household income: $50 k-$100 k	.89	.59	1.34	.93	.70	1.22
Gender: Cisgender man (ref)						
Gender: Cisgender woman	.56^**^	.40	.80	.84	.65	1.08
Gender: Trans identities^1^	1.71	.55	5.32	2.84^*^	1.13	7.16
LGBT2Q + identity: (Yes/unsure^2^)	1.28	.77	2.12	1.69^**^	1.15	2.49
Non-racialized						
Racialized (non-Indigenous)	1.21	.82	1.80	.90	.67	1.21
Indigenous	1.97^*^	1.01	3.86	.94	.50	1.74
Pre-existing mental health: Yes	2.67^**^	1.85	3.84	2.00^**^	1.50	2.65
Class (Class 3 is norm/ref)						
Class 1 (most at risk)	4.64^**^	3.19	6.75	2.84^**^	2.12	3.80
Class 2 (mixed risk)	1.22	.63	2.36	1.88^**^	1.25	2.82
Class 4 (most protected)	.50^*^	.28	.92	.45^**^	.30	.67

^1^ This includes participants who identified as trans women, trans men, non-binary, and Two-Spirit

^2^ Participants who indicated “unsure” when asked if they identified as LGBTQ2 + were included here to capture individuals who may be questioning their sexual orientation and/or holding fluid sexual identities

^*^p < .05

^**^p < .01

## Discussion

This cross-sectional survey study examines how patterns of co-occurring positive and negative emotions relate to mental health and structural vulnerability in the context of COVID-19. At a broad level, negative emotions were highly prevalent in this study, with the norm/reference class – representing 50.5% of the total sample – having moderate probabilities of endorsing negative emotions and low probabilities of endorsing positive emotions. This finding is supported by other studies conducted since the pandemic, including those showing quite high levels of distress and few positive emotions due to the broad health and social impacts of COVID-19 and associated public health protections [1–4]. While a public health concern in and of itself, this study also underscores that there is heterogeneity and inequity in emotional response patterns to the pandemic. Specifically, the latent class analysis identified a 4-class solution including a most at-risk class – representing 15.6% of the total sample – that experienced a combination of high probability of endorsing negative emotions and a low probability of endorsing positive emotions. This class disproportionately included adults with pre-existing mental health conditions and other socio-demographic characteristics that are indicative of intersecting structural vulnerabilities, rendering this group at inequitable risk for the psychosocial impacts of the pandemic. The emotional response patterns and mental health-related characteristics (e.g., current mental health, overall coping) of the most at-risk class stood in contrast to the most protected class, whose members had a low probability of endorsing negative emotions and a high probability of endorsing positive emotions, were more likely to report coping well and positive mental health, and had lower odds of suicidal ideation and increased used of substances to cope. Taken together, this evidence provides critical direction for informing equity-oriented mental health promotion efforts during and beyond the COVID-19 pandemic.

This study’s latent class analysis identified a most at-risk group of people, who could broadly be characterized as having a high probability of endorsing negative emotions combined with a low probability of endorsing positive emotions during a time of rising COVID-19 case numbers in Canada in January 2021. More specifically, this most at-risk class included a disproportionate number of participants who were younger (aged 18–34 years), in lower income categories, cisgender women, and LGBTQ2 + , particularly when compared to the norm/reference class and the most protected class. Roughly one in three (37.4%) members of this class endorsed having a pre-existing mental health condition and many more reported poor sleep, worse coping, and deteriorated mental health since the pandemic began. Membership in the most emotionally at-risk class was also associated with higher odds of experiencing suicidal ideation and increased use of substances to cope as a result of the COVID-19 pandemic in the past 2 weeks. These findings corroborate mounting evidence from international studies and knowledge syntheses signalling that the COVID-19 pandemic is having far-reaching yet unevenly distributed impacts on population mental health, with groups who experience intersecting structural vulnerabilities most affected [37, 40–42]. Findings also mirror epidemiological data from Canada during the first year of the pandemic [38], as well as longitudinal studies from other countries, including a United Kingdom-based analysis showing that women, young people (18–29 years), people from more socially disadvantaged backgrounds, and those with pre-existing mental health problems are being hardest hit by the mental health consequences of COVID-19, as measured across a range of factors including suicidal ideation [52]. Judicious monitoring of these significant and protracted mental health impacts and the underlying inequities implicated therein is essential for informing tailored public health responses, needed immediately and over the long term.

Supporting population mental health and emotional wellbeing requires a multi-pronged approach, inclusive of interventions across socio-ecological domains (i.e., from the individual through population levels) [38, 47]. This study identifies that having a combination of high positive emotions (e.g., hopefulness, calm) and low negative emotions (e.g., stress, sadness) was associated with reduced risk for adverse mental health outcomes (suicidal ideation and increased use of substances to cope) amid COVID-19, even after controlling for sociodemographic characteristics and pre-existing mental health conditions. At individual and service delivery levels, these findings underscore the need for action to support people experiencing predominantly negative emotions and limited positive emotions in response to the COVID-19 pandemic. Of relevance here are policy and practice interventions that align with psychoeducation and mental health literacy approaches, such as providing guidance and recommendations about how individual behaviours (e.g., media consumption, exercise) may influence emotions and experiences of stress; providing and scaling up access to safe, tailored counselling supports (e.g., services that are youth-oriented and queer- and trans-affirming); and more generally normalizing help-seeking and peer support, especially in times of hardship [11, 38, 53]. Reflecting on the study’s findings, these and other mental health promotion efforts should prioritize fostering more positive, deliberate, and engaged supports to help individuals manage pandemic-related stress. This is needed because, following Kavčič and colleagues [54], individual coping profiles and strategies are differentially associated with measures of mental health. Better outcomes have been tied to frequent use of approach-oriented strategies (e.g., active coping, instrumental and emotion support, planning, positive reframing, acceptance) and relative absence of avoidance strategies, such as denial and disengagement [55, 56]. To this point, another latent class analysis identified three distinct profiles of coping with COVID-19, with members of the engaged profile – characterized by active coping, planning, acceptance, and positive reframing – reporting the highest levels of wellbeing and lowest levels of stress and anxiety [54]. The current study substantiates the importance of understanding emotional response patterns and approaches to coping amid the pandemic, along with how these experiences relate to structural vulnerability and worsening mental health. Future work should seek to explicate the ways in which public health and policy action may promote uptake of more adaptative forms of coping with large-scale stressors and their inequitable impacts on population mental health.

Psychoeducational policy and practice efforts alone are insufficient for promoting and safeguarding population mental health, especially in contexts of crisis and inequity. Indeed, a comprehensive population health approach inclusive of mental health promotion, prevention, and treatment is needed to meaningfully and effectively respond to the population-level mental health burden of COVID-19 [38, 47]. This must include concerted action to address upstream causes of social and emotional stress, including underlying inequities (e.g., poverty, intersecting forms of oppression) implicated in the structural vulnerabilities surfaced in the current investigation. Already, there have been several calls for policy makers to take a public health and social determinants-based approach to mitigating the mental health impacts of COVID-19, while more generally fostering healthier and more equitable communities throughout and following the pandemic [35–37]. This approach could be advanced through several avenues, including strong and proactive support from governments against economic stressors [11, 57], such as enhancing access to safe and affordable housing, and instituting protections against unemployment and unjust loss of income (e.g., sick leave, gender pay gaps). Other interventions within an equity-oriented mental health promotion paradigm include support for grassroots, community-led responses to the pandemic, as well as investments in improving measurement and monitoring of intersections between COVID-19, mental health inequities, structural racism, and other forms of oppression [35, 36]. Also needed is enhanced access to mental health supports and prevention programs, including services that are geared toward and tailored to the needs of populations who face barriers accessing care (e.g., youth, LGBTQ2 + people, individuals living in rural communities), such as telehealth and/or community-specific services [37]. It is imperative that population-level mental health and equity considerations be prioritized when strategizing public health responses to the significant and unevenly distributed impacts of the COVID-19 pandemic.

This study has several strengths and limitations. This cross-sectional study was designed to include participation of people with diverse backgrounds and succeeded at yielding a large sample that is nationally representative according to age, gender, household income, and region. However, the sample may not be as representative according to other characteristics, including race, ethnicity, and ability. Likewise, the online survey format of this study may have contributed to the underrepresentation of individuals with limited access to technology. Additionally, the small sample size of certain groups (e.g., transgender people) and our use of a single survey item capturing all LGBTQ2 + identities hindered capacity for key subgroup analyses. From a design perspective, it is also important to note that the cross-sectional nature of the study prevents us from making causal conclusions regarding the directional nature of the associations reported in the findings. Additionally, although our assessments of emotions, suicidal ideation, and use of substances to cope explicitly asked participants to reflect on experiences felt as a result of the COVID-19 pandemic, it is possible that the observed differences on these measures may also reflect differences that existed prior to the pandemic. Survey responses may also have been influenced by emotions and stressors associated with the winter holidays after which this study was conducted. Nonetheless, this investigation provided a nuanced examination of how emotional response patterns at a period of rising case numbers during the COVID-19 pandemic are associated with structural vulnerability and mental health; longitudinal studies may help to further explore the directionality of associations and contextualize these trends. Of note, there may be some self-reporting and social desirability bias stemming from the survey’s use of self-reported measures for emotional responses, mental health, and using substances to cope. The inclusion of single-item measures for mental health here may be considered a study limitation, though such measures are valuable in applied public health research and have demonstrated associations with multi-item measures [58]. Still, future studies may benefit from leveraging multi-item, validated scales to examine pandemic-related emotional responses patterns and mental health experiences, whether separately or alongside single-item measures.

## Conclusion

This study characterizes population-level emotional response patterns in the context of the COVID-19 pandemic in Canada, identifying that structurally vulnerable subgroups of adults were most likely to experience negative emotions and least likely to experience positive emotions, while also being at higher risk for adverse mental health outcomes in the form of suicidal ideation and increased use of substances to cope. Evidence of the significant heterogeneity and inequity in how people are responding emotionally to the pandemic is compelling and important for informing public health responses to bolster population mental health alongside COVID-19. Specifically, findings from this study underscore the need for targeted psychoeducational efforts to promote more adaptive and positive forms of coping with pandemic-related stress, coupled with concerted public health action to promote and protect population mental health at a systemic level. Here, emphasis must be placed on multi-pronged policy and practice interventions that prioritize equity considerations, including those that are responsive to upstream drivers of structural vulnerability (e.g., intersecting oppressions) and that seek to foster collective mental health and wellbeing throughout and following the pandemic.

## Data Availability

The datasets analysed during the current study are available from the corresponding author on reasonable request.

## Abbreviations

AIC: Akaike information criterion
BIC: Bayesian information criterion
CI: Confidence interval
LGBTQ2 +: Lesbian, gay, bisexual, transgender, queer, Two-Spirit, and other sexual and gender minority
LMR-LRT: Lo-Mendel-Rubin likelihood ratio test
OR: Odds ratio
SSABIC: Sample size-adjusted BIC
